# A Randomized Controlled Trial on the Management of Post-COVID-19 Olfactory Dysfunction in a Pediatric Population

**DOI:** 10.7759/cureus.87026

**Published:** 2025-06-30

**Authors:** Nanki Hura, Pooja D Reddy, Amber Shaffer, Amanda Stapleton

**Affiliations:** 1 Department of Otolaryngology, University of Pittsburgh Medical Center, Children’s Hospital of Pittsburgh, Pittsburgh, USA; 2 Department of Otolaryngology, University of Pittsburgh School of Medicine, Pittsburgh, USA

**Keywords:** budesonide irrigations, olfactory training, pediatric covid-19, post-covid anosmia, smell rehabilitation

## Abstract

Background: Olfactory impairment is an important sequela of SARS-CoV-2 infection with potential long-term implications. Of treatments posited for post-COVID-19 olfactory dysfunction (OD), the strongest evidence supports olfactory training (OT) and topical steroids; however, a direct comparison of these treatments has yet to be published. Our objective was to directly compare the efficacy of OT versus budesonide irrigations for the treatment of post-COVID-19 OD in a pediatric population.

Methods: Pediatric patients aged six to 21 years presenting to the University of Pittsburgh, a tertiary children’s hospital in Pittsburgh, Pennsylvania, between September 2021 and May 2023 with at least eight weeks of smell dysfunction following COVID-19 infection were eligible. Participants were randomized (parallel, non-blinded) to 1) OT or 2) OT with budesonide irrigations (OT+BI) for eight weeks. Participants completed a University of Pennsylvania Smell Identification Test (UPSIT) and 22-item Sinonasal Outcome Test (SNOT-22) upon enrollment and eight-week follow-up.

Results: Twenty participants (75% female, median 16.0 years, range 7-19 years) were included, with 11 participants in the OT arm and nine in the OT+BI arm. The median time from COVID-19 infection to clinic presentation was 279.5 days (range 60-952 days). The OT arm did not show significant improvement in SNOT-22 or UPSIT scores at the eight-week follow-up, with limited follow-up data for the OT+BI irrigation arm.

Conclusions: We present the first randomized clinical trial comparing OT and topical steroids for the management of post-COVID-19 OD in a pediatric population. Although the participants did not show significant improvement in olfactory outcomes at eight weeks, hypothetically, longer treatment times may be warranted. This study was limited by a small sample size and high attrition, particularly in the intervention arm, which restricts the strength of our conclusions.

## Introduction

Olfactory dysfunction (OD) is a significant sequela of SARS-CoV-2 infection in adults and children, although pediatric data on prevalence, management, and long-term implications remain limited. A 2021 systematic review of 18 studies by Yan et al. reported a pooled prevalence of OD in children with COVID-19 of 15.97% [1]. While some children recover within 10 days, prolonged OD warrants further investigation [2,3]. Extrapolating from adult literature, patients with persistent chemosensory dysfunction are at significantly increased risk of developing depression and neurocognitive disorders, as well as decreased nutrition and increased frailty and mortality [4-7]. In children, such dysfunction may pose lifelong risks, potentially impacting growth, cognitive development, and overall quality of life. As COVID-19 has transitioned into an endemic phase, understanding and treating refractory OD in pediatric populations becomes increasingly urgent.

The pathophysiology of COVID-19-related OD involves viral infection of the sustentacular cells of the olfactory epithelium, leading to functional impairment [8]. In more severe or prolonged cases, damage to the olfactory receptor neurons and inflammatory processes in the olfactory mucosa may also contribute to sustained OD. When it comes to the treatment of OD, most of the evidence is derived from adult studies, and olfactory training (OT) and topical corticosteroids are currently the most widely supported interventions for post-viral OD [9]. Corticosteroids may combat local inflammation in the nasal mucosa and directly enhance olfactory function by modifying the activity of sodium-potassium adenosine triphosphatase in olfactory receptor neurons, a critical component in their function [10,11]. Several studies have explored the efficacy of corticosteroids in COVID-19-related OD. For example, Singh et al. investigated the effects of fluticasone and triamcinolone for anosmia and dysgeusia and reported significant improvements in sensory function after five days of treatment [12]. On the other hand, Abdelalim et al. evaluated the use of mometasone nasal spray in a randomized clinical trial for patients with recent COVID-19-related anosmia and found no statistically significant difference in olfactory outcomes between patients treated with mometasone and those receiving OT alone [13].

The role of OT in treating OD is an area of growing interest. Although the mechanism of OT is not fully understood, it has been paralleled to physical therapy for the brain, leveraging neural plasticity to aid recovery. Rezaeyan et al. demonstrated structural reorganization in olfactory processing areas following OT in post-traumatic adults using MRI [14]. Hummel et al. were among the first to show the efficacy of OT in improving olfactory function, with patients sniffing odorants such as rose, eucalyptus, lemon, and cloves twice daily for 12 weeks [15]. Since then, numerous post-viral OT studies have confirmed its clinical benefit, with Kattar et al.’s meta-analysis concluding that OT is associated with significant improvements in post-viral OD [16]. Despite these findings, OT has been minimally studied in pediatric populations, and there are no exclusive pediatric randomized controlled trials on OT efficacy.

The utility of both OT and topical corticosteroids for post-viral OD is actively being explored within the adult population. A randomized control trial by Nguyen and Patel demonstrated that a greater proportion of patients who underwent budesonide irrigations with OT had significant improvement in olfactory function compared to those who underwent saline irrigation with OT [17]. However, no studies have yet directly compared OT and topical steroids in pediatric populations with post-COVID-19 OD, creating a substantial gap in the literature. A 2024 prospective study by Chan et al. found that OT alone improved odorant identification in pediatric patients, suggesting OT's potential utility in children, though further research is needed [18].

Given the lack of pediatric-specific data and the potential developmental impacts in children, we conducted a randomized controlled trial to assess the efficacy of OT combined with BI in pediatric patients. We hypothesized that pediatric patients receiving OT with budesonide irrigations would experience greater olfactory improvement compared to those receiving OT alone. By directly comparing these two approaches, this study aims to inform and refine clinical decision-making for managing post-COVID-19 OD in children.

## Materials and methods

Study design

Reporting was completed in accordance with the Consolidated Standards of Reporting Trials (CONSORT) guidelines (Table 1) [19]. A randomized control trial with parallel treatment arms was conducted in September 2021-May 2023 following University of Pittsburgh Institutional Review Board approval (STUDY21050012) and was registered on ClinicalTrials.gov (NCT04964414). All patients presenting to the otolaryngology clinic at the University of Pennsylvania, a tertiary children’s hospital in Pittsburgh, Pennsylvania, with a chief complaint of “loss of smell” were screened for study eligibility. Inclusion criteria were ages six to 21 years old, no pre-existing sinonasal disease, clinical history of COVID-19 infection, and ≥8 weeks of smell dysfunction. Age six was chosen as the lower limit based on the FDA approval of nasal budesonide spray for children ≥6 years and improved reliability of olfactory self-reporting and University of Pennsylvania Smell Identification Test (UPSIT) performance at this age [20]. Smell dysfunction was defined as patient-reported persistent smell loss for ≥8 weeks and corroborated by the UPSIT score. Exclusions were duration of anosmia/dysosmia <60 days, previous olfactory retraining, prior interventions for loss of smell (excluding fluticasone nasal spray and azelastine nasal spray), any contraindication to nasal budesonide treatment, prior head trauma, congenital anosmia, history of brain tumor, neurocognitive disorders, multiple sclerosis, seizure disorder, cystic fibrosis, primary ciliary dyskinesia, or history of nasal polyps. Prior to participation, one parent or guardian consented for participants <18 years old, and participants ≥18 consented for themselves. Demographic data were collected for all participants. At the initial consult visit, participants performed the UPSIT and the Sino-Nasal Outcome Test-22 (SNOT-22) survey [20,21].

**Table 1:** CONSORT 2010 Checklist

Section/topic	Item no.	Checklist item	Reported on page no.
Title and abstract			
	1a	Identification as a randomised trial in the title	1
	1b	Structured summary of trial design, methods, results, and conclusions (for specific guidance see CONSORT for abstracts)	1
Introduction			
Background and objectives	2a	Scientific background and explanation of rationale	4-6
	2b	Specific objectives or hypotheses	6-7
Methods			
Trial design	3a	Description of trial design (such as parallel, factorial) including allocation ratio	8
	3b	Important changes to methods after trial commencement (such as eligibility criteria), with reasons	NA
Participants	4a	Eligibility criteria for participants	8
	4b	Settings and locations where the data were collected	8
Interventions	5	The interventions for each group with sufficient details to allow replication, including how and when they were actually administered	9-10
Outcomes	6a	Completely defined pre-specified primary and secondary outcome measures, including how and when they were assessed	9-10
	6b	Any changes to trial outcomes after the trial commenced, with reasons	NA
Sample size	7a	How sample size was determined	11
	7b	When applicable, explanation of any interim analyses and stopping guidelines	NA
Randomisation:			
Sequence generation	8a	Method used to generate the random allocation sequence	8
	8b	Type of randomisation; details of any restriction (such as blocking and block size)	8-9
Allocation concealment mechanism	9	Mechanism used to implement the random allocation sequence (such as sequentially numbered containers), describing any steps taken to conceal the sequence until interventions were assigned	8
Implementation	10	Who generated the random allocation sequence, who enrolled participants, and who assigned participants to interventions	8-9
Blinding	11a	If done, who was blinded after assignment to interventions (for example, participants, care providers, those assessing outcomes) and how	NA
	11b	If relevant, description of the similarity of interventions	NA
Statistical methods	12a	Statistical methods used to compare groups for primary and secondary outcomes	10
	12b	Methods for additional analyses, such as subgroup analyses and adjusted analyses	NA
Results			
Participant flow (a diagram is strongly recommended)	13a	For each group, the numbers of participants who were randomly assigned, received intended treatment, and were analysed for the primary outcome	11-12
	13b	For each group, losses and exclusions after randomisation, together with reasons	11-12
Recruitment	14a	Dates defining the periods of recruitment and follow-up	11
	14b	Why the trial ended or was stopped	12
Baseline data	15	A table showing baseline demographic and clinical characteristics for each group	17
Numbers analysed	16	For each group, number of participants (denominator) included in each analysis and whether the analysis was by original assigned groups	11-12, 17
Outcomes and estimation	17a	For each primary and secondary outcome, results for each group, and the estimated effect size and its precision (such as 95% confidence interval)	11-13
	17b	For binary outcomes, presentation of both absolute and relative effect sizes is recommended	NA
Ancillary analyses	18	Results of any other analyses performed, including subgroup analyses and adjusted analyses, distinguishing pre-specified from exploratory	NA
Harms	19	All important harms or unintended effects in each group (for specific guidance see CONSORT for harms)	NA
Discussion			
Limitations	20	Trial limitations, addressing sources of potential bias, imprecision, and, if relevant, multiplicity of analyses	15-16
Generalisability	21	Generalisability (external validity, applicability) of the trial findings	14-16
Interpretation	22	Interpretation consistent with results, balancing benefits and harms, and considering other relevant evidence	14-16
Other information			
Registration	23	Registration number and name of trial registry	2
Protocol	24	Where the full trial protocol can be accessed, if available	NA
Funding	25	Sources of funding and other support (such as supply of drugs), role of funders	NA

Treatment arms

The participants were randomized by the research coordinator/statistician to olfactory training (OT) for eight weeks or OT with budesonide irrigations (OT + BI) for eight weeks using a random sequence generator [22]. Stratified randomization was used to ensure equal proportions of patients in each group who had reasonable suspicion of COVID-19 infection without positive confirmation via testing. This study was not blinded, as the intervention protocols (nasal irrigation vs. OT alone) were visually and experientially distinct, precluding participant and clinician blinding. The attending pediatric otolaryngologist enrolled participants. One participant was placed in a treatment arm (OT + BI) different than what was randomly assigned (OT) at the initial clinic visit. This reassignment occurred due to clinical discretion as the participant had significant allergic symptoms and the treating physician deemed budesonide irrigations medically indicated.

The first treatment arm of OT alone consisted of choosing four scents each week and smelling each item for 60 seconds very close to the nose once daily. The four scents did not need to be new, discrete scents each week. Participants in the second treatment arm were additionally advised to perform budesonide irrigations once daily by adding 0.5 mg/2.0 mL of budesonide into a 200 mL saline sinus irrigation bottle and irrigating the sinonasal cavity. The specific odors were not standardized and were selected by participants weekly to enhance engagement, which aligns with prior olfactory training protocols encouraging personalization [15,16]. Each participant was provided a Smell Diary to be completed every day. The child and/or parent were instructed to record four scents each week with which to perform olfactory training and to checkmark each day that the four scents were smelled. Once weekly, the child was asked to rate their loss of smell on a scale from “no loss” (0) to “total loss” (10).

Data collection

Researchers called the parent or participant in weeks 3 and 5 and after completion of the initial eight-week period. Participants returned the Smell Diary at a scheduled follow-up appointment approximately eight weeks after enrollment, which was part of both routine clinical care and study protocol. The UPSIT and SNOT-22 were administered at all subsequent follow-up appointments. Participants unable to attend an in-person follow-up appointment were mailed the UPSIT and SNOT-22 to be completed and returned by mail. Adherence was quantified based on daily Smell Diary checkmarks, with percentage completion calculated. Missing data due to attrition were handled by excluding non-respondents from paired analyses. No statistical imputation or sensitivity analysis was performed due to the very small sample size.

Statistical analysis

Baseline characteristics and initial SNOT-22 and UPSIT scores were compared between the participants in the OT + BI and OT only groups using Fisher’s exact test, Wilcoxon rank-sum, or t-tests. SNOT-22 and UPSIT scores at eight-week follow-up were not compared between OT + BI and OT only groups due to significant loss to follow-up. SNOT-22 and UPSIT scores at the eight-week follow-up were compared with those at the initial visit using Wilcoxon signed-rank or McNemar’s Chi-squared tests; patients who were lost to follow-up were excluded from these paired analyses. Analyses were conducted using Stata/SE 16.1 (StataCorp, College Station, TX), with p < 0.05 considered significant.

## Results

Demographics and characterization of chemosensory dysfunction

Of 54 screened patients, 20 were enrolled, with 11 in the OT group and nine in the OT + BI group. Fifteen were female (75.0%), and their ages ranged from seven to 19 years (median 16.0 years) (Table 2). The median number of days from COVID-19 infection to the date of initial consult visit was 279.5 days (range 60-952 days). Two participants (10%) had received the COVID-19 vaccine. At baseline, 70% endorsed both smell and taste dysfunction. One participant (5.0%) had a history of radiation, three (15.0%) had a history of allergic rhinitis, three (15.0%) had a history of environmental allergies, one (5.0%) had a deviated septum, and two (10.0%) were immunocompromised (one from a history of cancer with radiation therapy and the other from treatment with mycophenolate mofetil).

**Table 2:** Patient demographics *Primary compliant at the initial clinic visit OT: olfactory training; BI: budesonide irrigations; N/A: not applicable

	Overall (n = 20, %)	OT (n = 11, %)	OT + BI (n = 9, %)	OT vs. OT + BI (P-value)
Sex				1.0
Male	5 (25)	3 (27)	2 (22)	
Female	15 (75)	8 (73)	7 (78)	
Race				1.0
White	18 (90)	10 (91)	8 (89)	
Black	1 (5)	1 (9)	0 (0)	
Asian	0 (0)	0 (0)	0 (0)	
Declined	1 (5)	0 (0)	1 (11)	
Ethnicity				
Hispanic	0 (0)	0 (0)	0 (0)	N/A
Non-Hispanic	19 (95)	11 (100)	8 (89)	
Unspecified	1 (5)	0 (0)	1 (11)	
Primary complaint*				
Abnormal smell and taste	14 (70)	6 (55)	8 (89)	0.2
Abnormal smell only	6 (30)	5 (45)	1 (11)	
Symptom onset post-COVID-19				
Immediate	14 (70)	7 (64)	7 (78)	0.6
Delayed	4 (20)	2 (18)	2 (22)	
Unknown	2 (10)	2 (18)	0 (0)	
Medical history				
Allergic rhinitis	3 (15)	0 (0)	3 (33)	0.07
Environmental allergies	3 (15)	0 (0)	3 (33)	0.07
Immunosuppression	2 (10)	1 (9)	1 (11)	1.0
Deviated septum	1 (5)	0 (0)	1 (11)	0.5
History of radiation	1 (5)	0 (0)	1 (11)	0.5
Vaccinated against COVID-19				
Yes	2 (10)	2 (18)	0 (0)	0.6
No	3 (15)	2 (18)	1 (11)	
Unknown	15 (75)	7 (64)	8 (89)	

**Figure 1:**
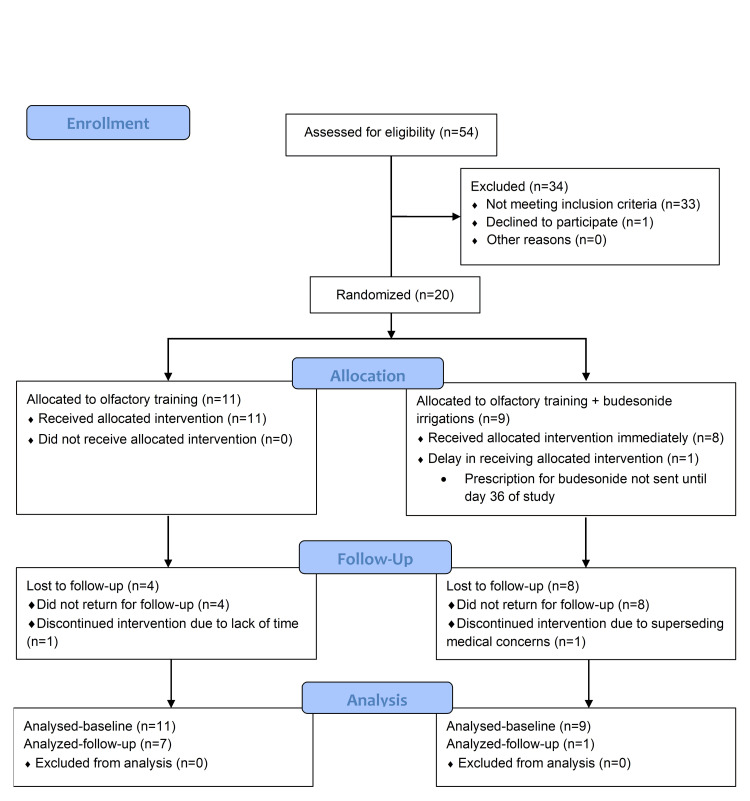
CONSORT diagram Consolidated Standards of Reporting Trials (CONSORT) flow diagram showing participant enrollment, allocation, follow-up, and analysis for both treatment groups: olfactory training (OT) and olfactory training with budesonide irrigations (OT + BI).

In the OT group, seven of 11 participants completed follow-up (mean adherence: 69%, range 30-100%). Four were lost to follow-up; one discontinued OT due to lack of time (Figure 1). In the OT + BI group, one of nine participants completed follow-up, reporting 91% adherence to irrigations and 80% adherence to OT. One withdrew due to cancer treatment; the other seven did not specify a reason. 

Smell Diary completion data

The seven participants in the OT-only group who returned for follow-up completed a mean of 69% (range 30-100%) of the prescribed smell retraining sessions based on the Smell Diary completion and returned at a mean of 10 weeks (range nine to 22 weeks). The one participant in the OT + BI group who returned for follow-up at 10 weeks reported completing budesonide irrigations 91% of days and 80% of smell retraining sessions.

SNOT-22 data

**Figure 2:**
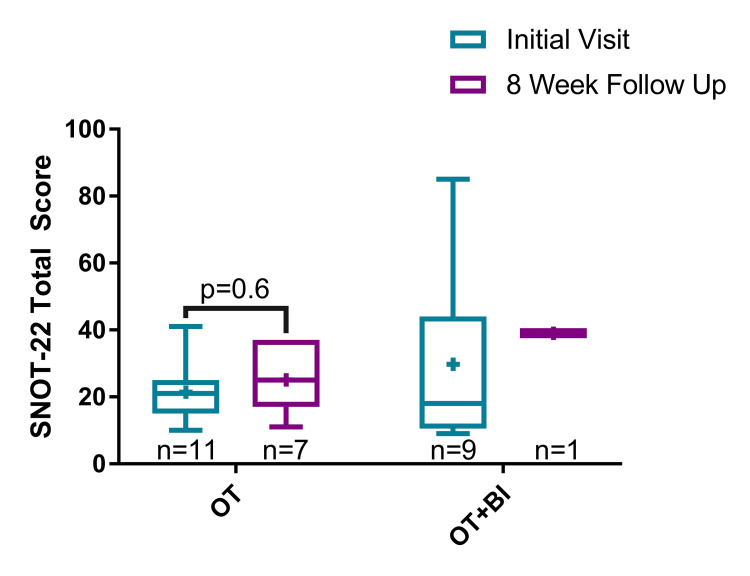
Total scores from the 22-item Sino-Nasal Outcome Test (SNOT-22) at the initial clinic visit and eight-week follow-up for both treatment arms: olfactory training (OT) and olfactory training with budesonide irrigations (OT + BI). SNOT-22: Sino-Nasal Outcome Test 22; OT: olfactory training; BI: budesonide irrigations; + indicates the mean.

For the OT-only arm, the median total SNOT-22 score at the time of initial clinic visit and eight-week follow-up was 21 (range 10-41) (n = 11) and 25 (range 11-37) (n = 7), respectively (Figure 2). There was no statistically significant difference in the total SNOT-22 scores between the initial clinic visit and eight-week follow-up for the OT-only arm (P = 0.6). For the OT + BI arm, the median SNOT-22 score at the initial clinic visit and eight-week follow-up was 18 (range 9-85) (n = 9) and 39 (n = 1), respectively.

UPSIT data

**Figure 3:**
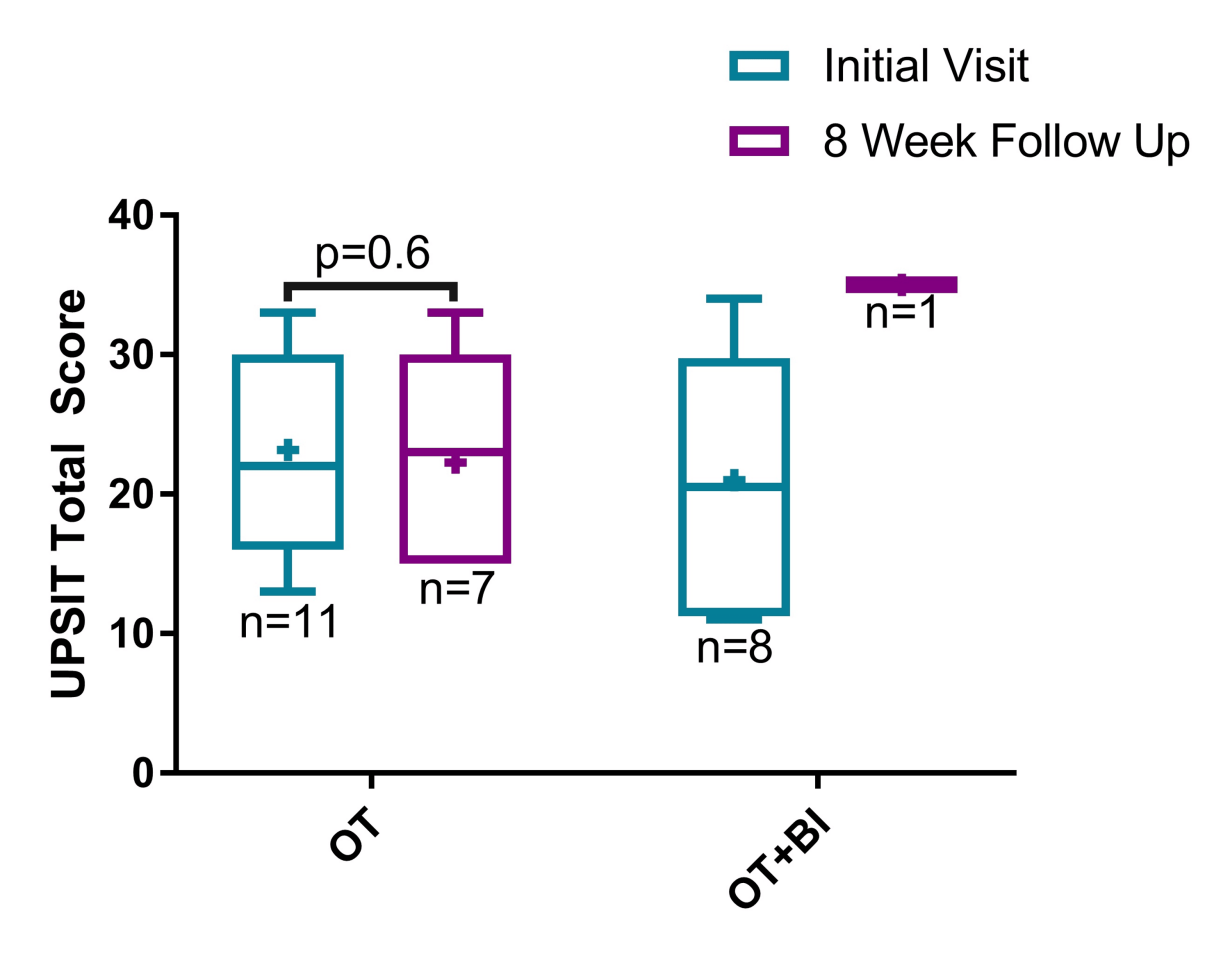
Total scores from the University of Pennsylvania Smell Identification Test (UPSIT) at the initial clinic visit and eight-week follow-up for both treatment arms: olfactory training (OT) and olfactory training with budesonide irrigations (OT + BI). UPSIT: University of Pennsylvania Smell Identification Test; OT: olfactory training; BI: budesonide irrigations; + indicates the mean.

For the OT-only arm, the mean ± standard deviation UPSIT score at the initial clinic visit and eight-week follow-up was 23.2 ± 6.9 (n = 11) and 22.3 ± 7.3 (n = 7), respectively (Figure 3). There was no statistically significant difference in the average UPSIT scores between the initial clinic visit and eight-week follow-up for the OT-only arm (P = 0.6). For the OT + BI arm, the mean UPSIT score at the initial clinic visit and eight-week follow-up was 21.0 ± 10.1 (n = 8) and 35.0 (n = 1), respectively. The odorants most frequently misidentified on the initial UPSIT were lime and soap (15/18, 83% misidentified for each). Due to the extremely limited follow-up in the OT + BI group, with only one participant completing post-treatment assessment, no meaningful comparative analysis between the two arms was feasible.

Study termination

The study was terminated before the target enrollment of 60 participants due to progressively slowing enrollment. No complications or adverse events were reported.

## Discussion

Of the many treatment options proposed for post-viral OD, the two modalities with the highest level of evidence in the literature are OT and topical steroids [9]. In this study, we did not observe significant improvements in SNOT-22 or UPSIT scores within the OT-only group at eight-week follow-up and were unable to conduct statistical analysis for the OT + BI group due to insufficient follow-up data; however, our findings provide insights into the challenges of managing persistent OD in the pediatric population. These findings should be considered exploratory due to the small sample size and substantial attrition.

Our demographic and clinical data further support our understanding of post-viral OD in younger patients. Consistent with prior studies, such as Yan et al., the majority of our participants were female, with a median age of 16 years. Seventy percent of participants reported both smell and taste dysfunction, highlighting the synergistic nature of the two systems. This high prevalence may suggest a need for more consideration of chemosensory rehabilitation when addressing OD. The median time from infection to presentation was nearly 280 days, highlighting the need for earlier identification and intervention in pediatric cases, which could improve treatment efficacy and outcomes. In addition, our findings emphasize the practical challenges inherent to pediatric trials, including participant dropout and difficulty in ensuring adherence to at-home therapies. The relatively involved nature of interventions like intranasal steroid irrigations and the younger age of the participants likely contributed to these challenges.

From the adult literature, there is no standardized protocol for performing OT, with some studies evaluating a short-term duration of OT, such as 12 weeks [15], and others studying longer courses up to 56 weeks [23]. However, it is noted that in a study by Konstantinidis et al. comparing a 56-week course of OT to a 16-week course, the longer-term training group was found to have significantly greater olfactory improvement, although both groups showed the most improvement in outcomes in the first 16 weeks. Several studies in adults have also shown that a shorter duration of OD is correlated with greater improvement in olfactory outcomes [17,24,25]. In our study, there was no objective or subjective improvement in olfactory outcomes after 8 weeks of OT; of note, the overall median time from COVID-19 infection to clinic presentation in our cohorts was 279.5 days. As such, it is possible that both a longer course of intervention and earlier intervention, in this same study group, may have shown improved efficacy. However, this is speculative, and future studies are needed to assess whether this is the case.

Limitations to this study include its largely homogenous sample (90.0% white) and small sample size, with participants lost to follow-up. Specifically, within the OT with budesonide irrigations group, the significant number of participants lost to follow-up limited the statistical analysis and thus the ability to compare study groups and draw conclusions. This missing data introduces a high risk of attrition bias and reduces confidence in the generalizability of our findings. As the majority of patients lost to follow-up did not provide a reason for discontinuing the study, we cannot determine whether dropout was related to treatment efficacy, tolerability, or other unmeasured factors that could have systematically influenced outcomes. We speculate that the reasons were likely multifactorial, perhaps in part due to the involved nature of the intervention, especially in regard to intranasal steroid application, the daily nature of the intervention, and the young age of the participants enrolled. We also likely enrolled fewer than our 60-participant target due to the relatively infrequent presentation of persistent post-COVID-19 loss of smell in the pediatric population and the waning nature of the pandemic due to vaccine availability. No imputation methods were applied, and per-protocol analysis was used; thus, our findings should be interpreted with caution and considered exploratory in nature. Additional limitations to the study included difficulty in ensuring treatment adherence to therapies administered in a home setting, especially in a pediatric population, and the delay between the time of COVID-19 infection and clinic presentation. As discussed above, a delay in presentation to the clinic is expectedly to result in a delay in the initiation of intervention, which may be critical in optimizing olfactory outcomes. Blinding was also not feasible given the visible differences in treatment protocols (nasal irrigations vs. scent exposure only), and we acknowledge this limitation in internal validity.

As the proportion of the pediatric population affected by long-term sequelae of COVID-19 infection, such as OD, only portends to increase with time, it is important that we continue to carefully consider and examine the treatment options that will be the safest, most convenient, and effective for these patients. We encourage those in the broader pediatric community to have a lower threshold for screening for post-viral OD, such that we may more quickly identify and thus initiate appropriate interventions for affected patients. Additional clinical comparison trials with larger sample sizes will be necessary to further assess the utility of adding intranasal steroid irrigations to a regimen of OT for the management of post-COVID-19 OD in pediatric patients.

## Conclusions

We performed the first randomized controlled trial comparing OT with and without topical steroid irrigations for the management of post-COVID-19 OD in a pediatric population. Although participants did not show significant improvement in olfactory outcomes at the eight-week follow-up, it is possible that longer treatment times may be efficacious; however, this is largely hypothetical. As a shorter duration of olfactory loss has been associated with improved olfactory outcomes in adults, earlier identification and intervention in pediatric patients may be associated with improved outcomes. Importantly, these findings serve as a pilot foundation for future larger-scale pediatric trials on OD.
